# Clinical Implementation of PSMA-PET Guided Tumor Response-Based Boost Adaptation in Online Adaptive Radiotherapy for High-Risk Prostate Cancer

**DOI:** 10.3390/cancers17172893

**Published:** 2025-09-03

**Authors:** Ruiqi Li, Mu-Han Lin, Nghi C. Nguyen, Fan-Chi Su, David Parsons, Erica Salcedo, Elizeva Phillips, Sean Domal, Aurelie Garant, Raquibul Hannan, Daniel Yang, Asim Afaq, MinJae Lee, Orhan K. Oz, Neil Desai

**Affiliations:** 1 Department of Radiation Oncology, The University of Texas Southwestern Medical Center, Dallas, TX 75235, USA; mu-han.lin@utsouthwestern.edu (M.-H.L.); fan-chi.su@utsouthwestern.edu (F.-C.S.); david.parsons@utsouthwestern.edu (D.P.); erica.salcedo@utsouthwestern.edu (E.S.); elizeva.phillips@utsouthwestern.edu (E.P.); sean.domal@utsouthwestern.edu (S.D.); aurelie.garant@utsouthwestern.edu (A.G.); raquibul.hannan@utsouthwestern.edu (R.H.); daniel.yang@utsouthwestern.edu (D.Y.); neil.desai@utsouthwestern.edu (N.D.); 2 Department of Nuclear Medicine, The University of Texas MD Anderson Cancer Center, Houston, TX 77030, USA; nghi.nguyen@mdanderson.org; 3 Department of Radiology, The University of Texas Southwestern Medical Center, Dallas, TX 75235, USA; asim.afaq@utsouthwestern.edu (A.A.); orhan.oz@utsouthwestern.edu (O.K.O.); 4 Department of Internal Medicine, University of Texas Health Sciences Center Medical School, Houston, TX 77030, USA; minjae.lee@uth.tmc.edu

**Keywords:** PSMA-PET, adaptive radiotherapy, stereotactic ablative radiotherapy, prostate cancer, MR-linac

## Abstract

Purpose or Objective: To evaluate the feasibility and clinical utility of integrating sequential PSMA-PET imaging into an offline–online adaptive workflow for response-based dominant intraprostatic lesion (DIL)-boosting high-risk prostate cancer treated with stereotactic ablative radiotherapy (SABR). Materials and Methods: As part of a prospective trial, patients were treated on MR- or CBCT-guided adaptive radiotherapy (ART) systems with prostate/pelvic node 5-fraction SABR (36.25 Gy/25 Gy) with DIL boost (50 Gy). Whereas traditional DIL boost volumes delineate full pre-therapy imaging-defined disease (GTVinitial), this study serially refined DIL boost volumes based on treatment response defined by PSMA-PET scans after neoadjuvant androgen deprivation therapy (nADT, GTVmb1) and fraction 3 SABR (GTVmb2). DIL delineation employed PET-PSMA fusion to CT/MR simulation and was guided by a rule-based %SUVmax threshold approach. Comparisons of GTV volumes and OAR dosimetry were performed between plans using GTVinitial versus GTVmb1/GTVmb2 for DIL boost, for each of the initial cohorts of five patients from the initially treated cohorts. Results: Five patients treated on MR-Linac (*n* = 3) or CBCT-based ART (*n* = 2) were analyzed. Three patients exhibited complete imaging response after nADT, omitting GTVmb boosts. Offline GTVmb refinements based on PSMA-PET were seamlessly integrated into ART workflows without introducing additional treatment time. DIL GTV volumes significantly decreased (*p* = 0.03) from an initial mean of 11.4 cc (GTVinitial) to 4.1 cc (GTVmb1) and 3.0 cc (GTVmb2). Dosimetric analysis showed meaningful reductions in OAR doses: rectal wall D0.035 cc decreased by up to 12 Gy, while bladder wall D0.035 cc and V18.3 Gy reduced from 52.3 Gy and 52.3 cc (Plan_initial) to 42.9 Gy and 24.9 cc (Plan_mb2), respectively. Urethra doses remained stable, with minor reductions. Sigmoid and femoral head doses remained within acceptable limits. Online adaptation efficiently addressed daily anatomical variations, enabling simulation-free plan re-optimization. Conclusion: PSMA-PET-guided adaptive microboosting for HRPCa SABR is feasible and effective. Standard MR-Linac and CBCT systems offer practical alternatives to BgRT platforms, enabling biology-driven dose personalization and potentially reducing toxicity.

## Simple Summary

High-risk prostate cancer is difficult to treat because tumors are often large and close to sensitive organs such as the bladder and rectum. Giving extra radiation to the most active tumor areas, called dominant intraprostatic lesions (DILs), may improve cancer control but often increase side effects. This study tested whether prostate-specific membrane antigen positron emission tomography (PSMA-PET) scans performed before and during treatment can guide changes in boost radiation volumes. We showed that tumors often shrink after hormone therapy and during early radiotherapy, allowing the boost volume to be reduced while still treating the entire prostate at the standard dose. This approach lowered the radiation dose to nearby organs without compromising coverage of the cancer. The findings suggest that response-guided boosting using widely available MR- or CBCT-guided adaptive radiotherapy systems is feasible and may improve the safety and adoption of focal dose escalation.

## 1. Introduction

High-risk prostate cancer (HRPCa) presents a significant clinical challenge due to an elevated risk of recurrence after definitive radiotherapy and androgen deprivation therapy (ADT) [1]. Focal radiation therapy (RT) dose escalation to dominant intraprostatic lesions (DILs) has been shown to improve disease control without significantly increasing toxicity. However, escalating the dose to focal volumes poses a challenge for organ-at-risk (OAR) sparing, particularly for critical structures such as the bladder, rectum, and urethra [2,3,4]. Adapting the DIL volume based on treatment response may allow for a more tolerable focal dose escalation without compromising tumor control. This is of particular relevance in HRPCa where initial DIL volumes are typically bulkier and where RT is preceded by cytoreductive neoadjuvant ADT (nADT). However, the traditional mpMRI definition of DIL volume is degraded by reduced discrimination after treatment with ADT or RT [5].

Prostate-specific membrane antigen (PSMA) positron emission tomography (PET) has emerged as a sensitive imaging modality for both PCa staging and for highly reproducible intraprostatic tumor delineation [6,7,8,9,10]. PSMA-guided DIL boost during RT for PCa has been described in multiple pilot studies [11,12] as having an acceptable toxicity profile at modest dose escalation but with concern for excess toxicity at higher DIL boosts. This has resulted in a current randomized phase III trial of PSMA-guided DIL boost electing a relatively low DIL boost (40–42 Gy/5fx), as compared to MR-guided DIL boost trials which escalated successfully to 50 Gy/5fx [13].

Serial PSMA-PET imaging during neoadjuvant ADT and treatment has the potential to quantify tumor response and refine target volumes accordingly. Early clinical studies have shown that over 60% of HRPCa patients exhibit substantial intraprostatic tumor shrinkage on PET following ADT and that residual PSMA-PET after 3 months of nADT correlated to the extent of histopathology response at surgery [5,14]. This opens the possibility of adapting boost volumes to exclude responding tissue, thereby enhancing OAR sparing while maintaining dose intensity to residual disease.

Such proposed biology-guided radiotherapy (BgRT) has gained attention for its potential to personalize treatment using real-time or interval biological imaging [15,16,17]. While current BgRT systems do not yet offer online adaptation, standard adaptive radiotherapy (ART) systems, such as CBCT-based platforms and MR-guided radiotherapy (MRgRT), enable the incorporation of multiple imaging modalities into ART workflows [18,19]. These capabilities allow incorporation of post-ADT and mid-treatment PSMA-PET scans for response-driven boost redefinition. In addition to it, the superior soft tissue visualization provided by MR-Linac systems facilitates pelvic adaptive workflow, making them ideal for the transition to response-guided treatment [20,21]. Incorporation of MRI into treatment machines for delivery of MR guided radiotherapy (MRgRT) has been well studied and was recently associated with lower acute urinary toxicity in one randomized trial of prostate SABR [22,23].

In this study, we present a practical and scalable workflow for integrating serial PSMA-PET imaging into standard ART platforms for HRPCa. By adapting the DIL boost volume at two key timepoints—after neoadjuvant ADT and mid-treatment—we implemented a biology-guided adaptive radiotherapy strategy using both MRgRT and CBCT-based systems. We evaluated the feasibility, dosimetric benefits, and operational integration of this response-based adaptive focal boosting strategy within routine clinical practice.

## 2. Methods

### 2.1. Study Design and Schema

**Figure 1:**
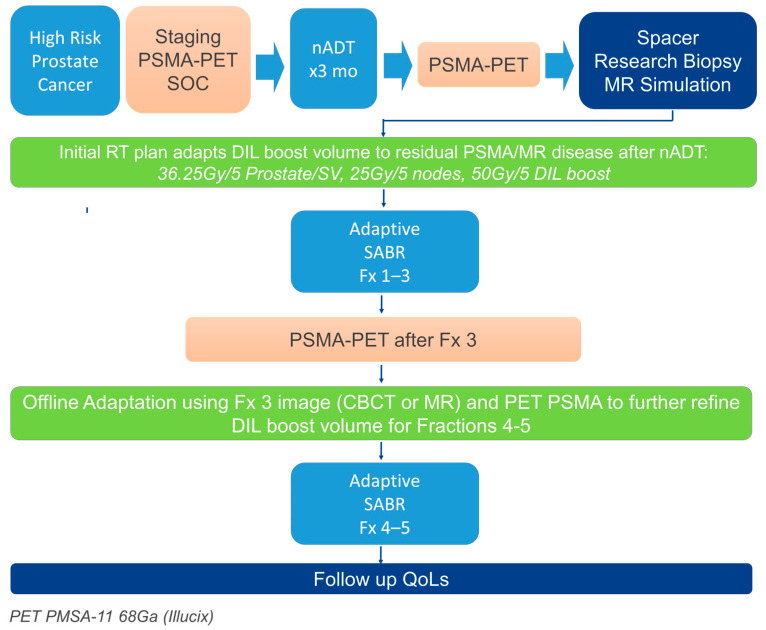
Trial scheme.

This technical study evaluated representative patients treated on an ongoing single-institution, IRB-approved phase 1B study (NCT06044857) evaluating PSMA-PET-guided adaptive SABR for men with high-risk prostate cancer (shown in Figure 1). Eligible patients had biopsy-proven HRPCa per NCCN criteria (≥cT3a, PSA > 20 ng/mL, or ISUP Grade Group 4–5) and underwent baseline 68Ga-PSMA-11 (Illuccix, Telix, Melbourne, Australia) PET/CT for staging. All patients received neoadjuvant ADT for 3 months prior to SABR and continued as tolerated for 18–24 months total. The SABR fractions for patients evaluated in this study were delivered every other day or weekly. Two subsequent PSMA-PET response scans were strategically timed to guide sequential adaptation of the DIL boost volume during therapy. Pre-therapy PET was acquired before the start of ADT; this scan defined the initial gross tumor volume (GTVinitial) and was used for initial target delineation and staging. Although not used for treatment planning, it provided a reference for disease burden prior to systemic therapy. The post-ADT PET was conducted after approximately 3 months of neoadjuvant ADT and prior to simulation. It was used to define the first response-adapted microboost target (GTVmb1), which targeted the adaptive treatment plan for the first three fractions (Fx 13). After the third fraction, a mid-treatment PET was performed typically within 1–3 days to allow time for assessment again for the adaptation to residual PSMA-avid disease following partial radiotherapy delivery. If persistent uptake was observed, the microboost target volume was redefined as GTVmb2, which would replace GTVmb1 and be used for the adaptive treatment for fractions 4 and 5. In cases of PSMA-PET complete response, boost was omitted while maintaining standard-of-care whole-gland coverage (36.25 Gy/5 fractions).

### 2.2. Target Volume Definition and Treatment Planning

In each PET scan, DILs were segmented using a consistent semi-automated thresholding approach (protocol guidance ~35%SUVmax for GTVinitial) following published approaches [13,24] and co-registered with simulation or on-treatment CT&MR images. Following treatment exposure, GTVmb1/2 thresholds in practice required modification according to residual SUVmax in a rule-based approach established early in the trial: 41% if SUVmax > 12, 55% if SUVmax 5.1–12.0, and 75% if SUVmax 3.0–5.0. Manual refinements ensured the PSMA-avid lesion remained within the prostate contour. Corresponding PTVinitial/mb1/mb2 were created without margin and subtracting bladder, rectum and urethra and prescribed to 50 Gy in 5 fractions. The prostate with at least 1 cm proximal seminal vesicles was treated to 36.25 Gy with 3 mm expansion, distal seminal vesicles (3 mm expansion) and pelvic nodes (5 mm expansion) to 25 Gy and optional nodal boost for PSMA-avid involvement to 35 Gy (0–3 mm expansion). Reference plans were created for each GTV definition. Primary OARs include rectal wall, bladder wall, small bowel, sigmoid colon, urethra, and femoral heads. Plans were created using either Monaco or Ethos treatment planning system (TPS), following trial-specific dose constraints (Table 1). Adaptation was performed offline for GTVmb1 and GTVmb2, with reference plans updated using the new contours and original beam weights and aperture shapes. Online ART then re-optimized segments per fraction, accounting for daily anatomy.

### 2.3. Integrated Offline and Online Adaptation Workflow

To support biologically guided dose escalation in a routine clinical setting, we implemented a hybrid offline–online adaptive workflow that integrates sequential PSMA-PET imaging into the ART process. Successful execution of this workflow required close interdisciplinary collaboration. Radiation oncologists and nuclear medicine physicians jointly reviewed PET scans and delineated response-adapted targets. The hybrid approach allows physicians to review new PET images and update the boost GTV volume contours without the time constraints of an online setting, thereby enhancing the safety, quality, and efficiency of treatment. This strategy also strikes a balance between treatment quality and clinical resource demands for ART.

**Figure 2:**
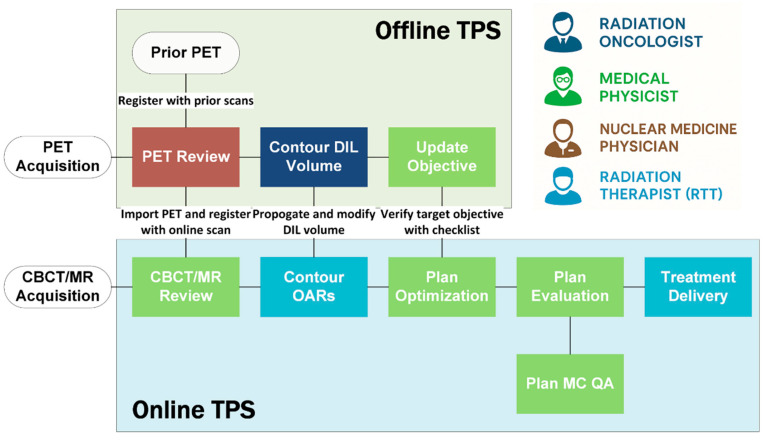
Workflow for PSMA-PET-guided adaptive radiotherapy incorporating sequential imaging and planning steps across offline and online treatment planning systems (TPSs). Role responsibilities are indicated by color-coded professional icons.

As illustrated in Figure 2, the workflow begins with offline PET fusion with simulation scans (MR or CT/MR) and DIL contouring based on biological response. Subsequently, the auto-contouring templates or optimization objectives are updated as needed. These updates are completed in advance of the next treatment fraction so that the modified parameters can be automatically incorporated into the online ART process, where new contours and objectives guide on-table re-optimization.

Daily adaptive treatment followed platform-specific workflows. For both MR-Linac and CBCT-Linac systems, patients were immobilized using a custom frame to ensure setup reproducibility. On the MR-Linac, a daily T2-weight MRI was acquired, and the newly contoured GTV structures were propagated to the onboard anatomy. Beam weights and segments were then re-optimized using the Monaco treatment planning system. On the CBCT-Linac, a daily CBCT was acquired. To ensure accurate and efficient propagation of the updated GTV contours, CBCT images were registered to the PET/CT reference images using implanted fiducial markers. This fusion step was critical for maintaining target fidelity in the presence of variable soft-tissue contrast.

To support safe and consistent implementation across both platforms, a dedicated adaptive physics log and structured communication protocol were maintained. Key workflow elements were documented using a standardized checklist, including reference plan identification, prescription and normalization parameters, critical organ-at-risk (OAR) objectives, and optimization guidance.

### 2.4. Evaluation Strategy

Workflow feasibility was first validated using phantom dry runs to assess image quality, registration, and response-adapted dose accumulation on both platforms. Clinical implementation was then tested on trial patients. Timing metrics, plan adaptation efficiency, and online decision-making were documented.

Retrospective dosimetric comparisons were conducted for Plan_initial (planned upon GTVinitial) versus Plan_mb1 (planned upon GTVmb1) and Plan_mb2 (planned upon GTVmb2), focusing on OAR dose metrics while maintaining reasonable target coverage: Rectal wall D_0.035cc_, Bladder wall D_0.035cc_ and V_18.3Gy_, Urethra D_0.035cc_, sigmoid D_0.035cc_ and V_32.5Gy_, and femoral head V_30Gy_. Changes in GTV boost volume across timepoints were assessed using Wilcoxon signed-rank tests. OAR dose reductions were analyzed by plan comparison. A *p*-value < 0.05 was considered statistically significant.

## 3. Results

### 3.1. DIL GTV Volume Tracking and Dosimetric Comparison

Five out of eight initially treated patients on either MR-Linac (*n* = 5) or CBCT-based (*n* = 3) systems were assessed to ensure platform-independent utility. Three patients showed complete response, thus the GTVmbs for dose escalation were omitted.

**Figure 3:**
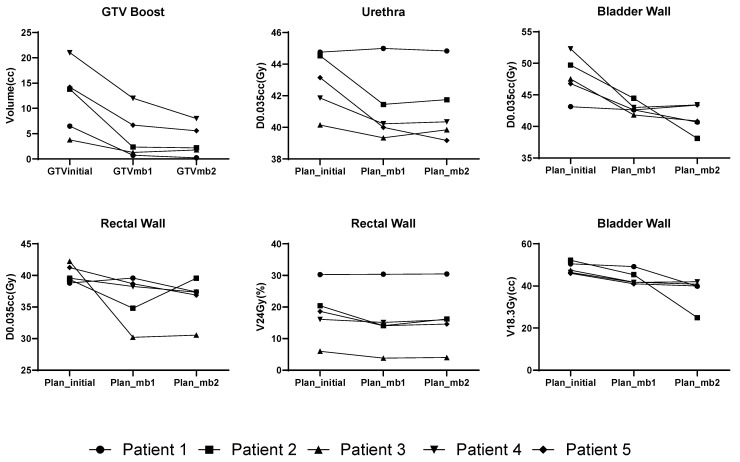
Individual patient-level trends in GTV boost volume and organ-at-risk (OAR) dose metrics across three adaptive planning stages: initial (Plan_initial), post-ADT (Plan_mb1), and mid-treatment (Plan_mb2).

We first track and evaluate the volume change in the DIL GTV volume, as shown in Figure 3. GTV boost volumes showed a substantially decrease (*p* < 0.05) for all patients from an initial mean of 11.4 cc (GTVinitial) to 4.1 cc (GTVmb1) and 3.0 cc (GTVmb2).

Individual patient-level trends in organ-at-risk (OAR) dose metrics across three plans are shown in Figure 3. Dosimetric comparisons showed improved V_50Gy_ coverage as well as meaningful reductions in all proximal OAR doses. Rectal wall D_0.035cc_ showed reductions of up to 12 Gy. Bladder wall D_0.035cc_ and V_18.3Gy_ also decreased significantly (*p* < 0.05), with the largest reduction from 52.3 Gy and 52.3 cc in Plan_initial to 42.9 Gy and 24.9 cc in Plan_mb2. Urethra doses remained stable, with D_0.035cc_ reduced by 3 Gy for one case. Sigmoid and femoral head doses remained within protocol constraints and did not vary meaningfully between plans. A complete summary of PTV coverage and OARs constraints across initial and adaptive plans is included in Table 1.

### 3.2. Workflow Feasibility and Plan Adaption

**Figure 4:**
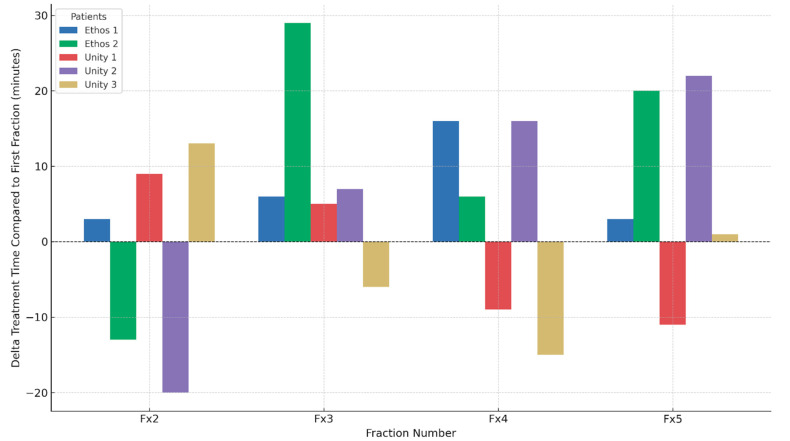
Bar plot illustrates treatment time differences for each fraction relative to the first fraction across five patients treated on Ethos (CBCT-Linac) and Unity (MR-Linac) platforms.

The adaptive workflow was implemented on both MR-Linac and CBCT-based ART platforms. All treatment fractions were successfully delivered without technical intervention. Figure 4 summarizes the change in treatment duration for each fraction relative to the first, across five patients. Statistical analysis using the Wilcoxon signed-rank test showed no significant difference in treatment duration compared to the first fraction for either platform (Unity: *p* = 0.85; Ethos: *p* = 0.078), suggesting overall workflow consistency despite adaptation. The median total treatment time for the fourth fraction—when the DIL volume was updated—was 78.4 min, compared to 84.2 min for all other fractions. Offline adaptive plan updates were generally completed within 30 min, including approximately 20 min for contour transfer and optimization, and 10 min for independent review.

**Figure 5:**
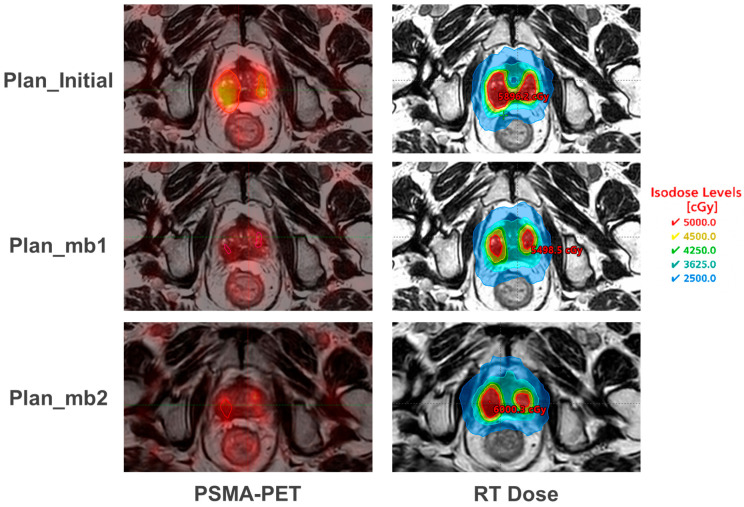
Representative axial images showing serial PSMA-PET signal and corresponding radiotherapy (RT) dose distributions for three adaptive boost plans: initial (Plan_initial), post-ADT (Plan_mb1), and mid-treatment response (Plan_mb2). (**Left column**) shows PSMA-PET/MRI fusion images with evolving dominant intraprostatic lesion (DIL) contours. (**Right column**) displays corresponding RT plans with isodose lines (2500–5000 cGy) illustrating progressive dose conformality and reduced high-dose exposure with GTVmb adaptation.

Axial images of one representative case are illustrated in Figure 5. The initial PSMA-PET scan revealed a 12 cc dominant lesion located adjacent to the urethra and posterior bladder base, presenting a potential challenge for focal dose escalation. Following 3 months of neoadjuvant ADT, repeat PET demonstrated a marked volumetric response, with the DIL shrinking to 4 cc. Based on this response, the treating physician elected to adapt the boost volume to the new post-ADT GTVmb1. After the third SABR fraction, a second PSMA-PET (PET2) was acquired and fused offline to both the fraction 3 MR image and the simulation MR using rigid alignment within the TPS. The radiation oncology physician delineated GTVmb2 from PET uptake in collaboration with nuclear medicine, following consensus guidelines on %SUVmax thresholds, together with MRI anatomical constraints. The new structure set was transferred back to the offline Monaco. The reference plan then was calculated using the original MU, only with adaptive margin structure replacing the original GTVmb1 and PTVmb1 and with update DVH constraints. Since the same plan, no reprint or any further chart preparation is needed. And the recalculated plan was used as the reference plan for the adaptation of the following fractions; for the next treatment, the fluence and segments were re-optimized to achieve the desired coverage while maintaining the OAR constraints.

## 4. Discussion

This study demonstrates the technical feasibility and potential clinical value of incorporating sequential PSMA-PET imaging into adaptive SABR workflows for high-risk prostate cancer. Our approach builds on emerging trial evidence supporting PSMA-PET-guided radiotherapy [25]. By adapting the DIL boost volume based on tumor response to neoadjuvant ADT and mid-treatment SABR delivery, we achieved meaningful reductions in target volume and organ-at-risk (OAR) dose without compromising coverage. In our cohort, average DIL volume decreased by 64% from baseline to post-ADT and by 74% by mid-treatment, which directly translated into improved bladder and rectal sparing—particularly in maximum point dose and intermediate dose volumes. These improvements are clinically meaningful, as rectal and bladder toxicities are dose-dependent and often limit focal dose escalation. Importantly, this adaptive approach maintained a minimum “floor” of dose at 36.25 Gy in five fractions, which has gained widespread implementation and comfort of use in prostate SABR across risk categories, thus mitigating the risk of overly aggressive dose de-escalation and associated local failure.

Importantly, our workflow demonstrates that biology-guided radiotherapy can be implemented using standard adaptive radiotherapy (ART) platforms, including MR-Linac and CBCT-based systems. The use of offline PET fusion and pre-contouring allowed for seamless integration into online adaptive sessions without additional simulation or approval burden. Retaining original beam weights and apertures from the reference plan reduced the need for reprinting, while re-optimization ensured appropriate target coverage. In comparison to standard replans which take days for plan optimization, MD review and approval, and chart preparation, the updated workflow reduces the total time to be within 3–40 min, which brings convenience and availability of the clinic. These efficiencies—along with the safety provided by a dedicated physics checklist and PET image review—suggest that adaptive PET integration is scalable in the clinic.

BGRT has an emerging interest in multiple treatment sites. The benefit is obvious as the PET scan is arguably the only scan that extends beyond the anatomic landmarks to the actual biomarkers of the patient [17]. The SCINTIX machine (RefleXion Medical, Inc., Hayward, CA, USA) is a hybrid radiotherapy platform that incorporates PET into the architecture of a ring-gantry linear accelerator system [26]. The benefit of PET-Linac is obvious; however, existing trials led to FDA approval for this machine to treat FDG-guided therapy in the lung and bone [27], with other disease sites to follow. However, the machine limitations still preserve to be an issue which includes size of target and plan quality [28]. Furthermore, while equipped with high-quality kV-CT, the PET-Linac platform currently does not allow adaptation availability, and an additional challenge includes the robustness against different lesion size defined by SUV intake.

Regardless of platform, optimal delineation of PET-based GTV rendering based upon PSMA avidity of a lesion remains critical and needs to consider treatment exposure. We utilized a %SUVmax approach due to established data for its reproducibility and sensitivity in the treatment naive setting [24] for the 68 Ga-PSMA-11 imaging agent used in this study. Currently, there are no established guidelines on the use of %SUVmax, but reports have used thresholds ranging from 20% to 60% of SUVmax: Spohn et al. used thresholds between 20 and 40% SUVmax in an PSMA-PET study, demonstrating high sensitivity and specificity for intraprostatic tumor delineation [29]. Goodman et al. applied a 23–40% SUVmax window in a PSMA-PET/CT radiotherapy planning feasibility study, showing the practicality of focal DIL boost [11]. Zhang et al. compared manual versus semi-automatic segmentation with thresholds from 30 to 60% SUVmax in a PET/MRI evaluation study, finding strong correlation across methods [30]. In the prospective PROBE Phase 2 trial, Singh et al. reported a median optimal threshold of ~48% SUVmax for PSMA-PET–guided SBRT boost [25]. Collectively, these findings emphasize the need for reproducible, rule-based SUVmax contouring guidelines to standardize adaptive boosting strategies and to define them according to lesion avidity and response to treatment. An important expected output of this study is to provide novel insight into the practical parameters for applying a thresholding approach in the midst of ADT and RT.

The proposed workflow provides an efficient alternative that can be readily implemented using existing ART systems. However, several limitations must be acknowledged. First, our study cohort is small, and only five patients had evaluable mid-treatment adaptation data. Second, the PSMA-PET imaging schedule, particularly PET timing after fraction 3, was selected for feasibility and may not capture optimal biologic response kinetics. Third, our analysis is for dosimetry only; long-term toxicity or oncologic outcomes are beyond its scope. It also should be noted that discrepancy was observed for the OAR volumetric metrics, especially for the intermediate dose falloff for both initial plan and adaptive plan between the MR-Linac and CBCT-Linac. These differences were not attributed to target volume, beam arrangement, or optimization parameters, but to the inherent distinctions in treatment delivery system: the MR-Linac lacks collimator rotation and uses a fixed couch with lower modulation capability, while the Ethos system features dual-layer MLCs and dynamic collimator motion, enabling higher modulation and improved dose shaping. Despite this, the MR-Linac offers advantages in soft-tissue visualization and real-time target tracking, which are integral to certain adaptive workflows. These system-specific tradeoffs should be considered when designing response-adaptive strategies based on available clinical infrastructure. At our institution, we plan to broaden enrollment in the ongoing trial to validate workflow efficiency and toxicity outcomes. In parallel, we are developing standardized protocols for PET/ART integration—including structured checklists and physics logs—to support reproducibility and quality assurance. These tools are intended for multi-institutional use to facilitate broader dissemination of this workflow.

## 5. Conclusions

PSMA-PET-guided adaptive microboosting for HRPCa SABR is feasible and effective. Standard MR-Linac and CBCT systems offer practical alternatives to BgRT platforms, enabling biology-driven dose personalization and reduced toxicity.

## Figures and Tables

**Table 1:** Summary of prescription dose coverage and organ-at-risk (OAR) constraints across Plan_initial, Plan_mb1 and Plan_mb2. Reported values are cohort means, with minimum–maximum ranges shown in brackets.

Prescriptions/Coverage Goals for PTVs					
Structure	Parameter	Objective	Plan_Initial	Plan_mb1	Plan_mb2
PTVPros3625_5	V36.25 Gy	≥98%	98 [93.3, 100]	98.0 [95.0, 100]	98.0 [91.3, 99.3]
PTVPelv2500_5	V25 Gy	≥98%	93.4 [88.0, 98.0]	94.0 [90.6, 97.5]	91.0 [88.6, 98.5]
PTVmb_5000_5	V50 Gy	≥90%	70.4 [27.8, 95.0]	75.4 [25.0, 95.0]	85.4 [45.0, 95.0]
**Normal Tissue/Organ-at-Risk (OAR) Constraints**					
Structure	Parameter	Objective	Plan_Initial	Plan_mb1	Plan_mb2
Rectal Wall	D0.035 cc	≤42.5 Gy	39.6 [38.8, 42.2]	38.2 [30.2, 39.6]	38.2 [30.2, 39.6]
	V39 Gy	<20%	1.1 [0.0, 2.1]	0.0 [0.0, 0.5]	0.0 [0.0, 0.5]
	V24 Gy	<50%	18.6 [6.0, 30.3]	14.1 [3.8, 30.4]	16.0 [4.0, 30.5]
Bladder Wall	V18.3 Gy	<50 cc	47.5 [46.0, 52.3]	41.8 [41.0, 49.2]	40.0 [25.0, 42.0]
	D0.035 cc	<42.5 Gy	47.5 [43.2, 52.3]	42.6 [41.8, 44.4]	40.8 [38.1, 43.4]
Bowel Small	D0.035 cc	<27.5 Gy	29.7 [26.5, 30.9]	28.9 [26.5, 32.1]	28.7 [26.2, 33.4]
	V25 Gy	<20 cc	4.0 [0.0, 5.1]	2.3 [0, 3.4]	2.1 [0, 3.2]
	V20 Gy	<30 cc	51.7 [49.9, 76.5]	42.9 [38.2, 67.6]	41.4 [35.6, 79.9]
Sigmoid	D0.035 cc	<37.5 Gy	29.7 [26.5, 30.9]	28.9 [26.5, 32.1]	28.7 [26.2, 33.4]
	V32.5 Gy	<20 cc	0.0 [0.0, 0.0]	0.0 [0.0, 0.0]	0.0 [0.0, 0.0]
Urethra	D0.035 cc	<42.5 Gy	43.2 [40.2, 44.8]	40.2 [39.3, 45.0]	40.4 [39.2, 44.8]
Femoral Heads	V30 Gy	<10 cc	0.0 [0.0, 0.0]	0.0 [0.0, 0.0]	0.0 [0.0, 0.0]

## Data Availability

The data presented in this study are available on reasonable request from the corresponding author. The data are not publicly available due to patient privacy and institutional restrictions.
